# Multifunctional Woven Structure Operating as Triboelectric Energy Harvester, Capacitive Tactile Sensor Array, and Piezoresistive Strain Sensor Array

**DOI:** 10.3390/s17112582

**Published:** 2017-11-09

**Authors:** Kihong Kim, Giyoung Song, Cheolmin Park, Kwang-Seok Yun

**Affiliations:** 1 Department of Electronic Engineering, Sogang University, 35 Baekbeom-ro, Mapo-gu, Seoul 04107, Korea; ekdrmsking@sogang.ac.kr; 2 Department of Materials Science and Engineering, Yonsei University, 50 Yonsei-ro, Seodaemun-gu, Seoul 03722, Korea; sjydanny@naver.com (G.S.); cmpark@yonsei.ac.kr (C.P.)

**Keywords:** smart fabric, triboelectric energy harvester, capacitive sensors, tactile sensors, strain sensors

## Abstract

This paper presents a power-generating sensor array in a flexible and stretchable form. The proposed device is composed of resistive strain sensors, capacitive tactile sensors, and a triboelectric energy harvester in a single platform. The device is implemented in a woven textile structure by using proposed functional threads. A single functional thread is composed of a flexible hollow tube coated with silver nanowires on the outer surface and a conductive silver thread inside the tube. The total size of the device is 60 × 60 mm^2^ having a 5 × 5 array of sensor cell. The touch force in the vertical direction can be sensed by measuring the capacitance between the warp and weft functional threads. In addition, because silver nanowire layers provide piezoresistivity, the strain applied in the lateral direction can be detected by measuring the resistance of each thread. Last, with regard to the energy harvester, the maximum power and power density were measured as 201 μW and 0.48 W/m^2^, respectively, when the device was pushed in the vertical direction.

## 1. Introduction

In recent years, wearable devices, which provide a connection between humans and devices anywhere and at any time, have been widely used in various fields such as health care, medical diagnosis, fitness, wellness, entertainment, and military applications to measure the physical conditions of the human body or to check body motion and activity in real time [1,2,3,4,5]. These devices are mainly implemented in the form of wearable accessories such as smart bands and smart glasses, body-attachable devices such as smart lens and smart tattoos, and smart clothes such as sportswear and shoes [6,7,8,9,10]. Among them, smart clothes are a wearable platform that can be equipped with sensors, actuators, data processors, and power sources and has the advantage of being worn like ordinary clothing [11,12].

According to the application and purpose of use, a variety of sensors including pressure sensors, strain sensors, temperature sensors, accelerometers, gyro sensors, gas sensors, and tactile sensors have been utilized in smart clothes [13,14,15,16,17]. Among them, tactile sensors, which detect tactile information through physical touch, have been widely developed to detect various types of information including shape, texture, softness, temperature, vibration, and shear or normal forces. Such a tactile sensor can be implemented by using capacitive, optical, piezoelectric, and piezoresistive transducers to detect the position and magnitude of the input touch force [18,19,20,21]. Capacitive transducers have been widely adapted for smart clothes applications because they can be relatively easily implemented in a large area in a textile structure. 

Lee et al. reported on a flexible and sensitive textile-based pressure sensor using conductive fibers coated with rubber materials [22]. Hasegawa et al. proposed a tactile sensor by weaving hollow tubes and demonstrated a capacitive tactile sensor array in a textile structure [23]. The strain sensors in smart clothes are used for healthcare systems such as body movement monitoring, medical monitoring, and sports and injury prevention in real time. When the human body deforms intentionally or unintentionally, the strain sensors monitor the magnitude, direction, frequency, and location of the deformation by measuring resistance changes. Wang et al. fabricated wearable strain sensors through a simple carbonization process with woven structures of silk fabric [24]. In addition, Li et al. and Park et al. reported on highly stretchable and flexible strain sensors composed of carbon-based materials and polymer matrices [25,26]. The devices detected human motion by mounting the sensors on the joint areas of the finger, elbow, and knee.

An important issue for these wearable devices and smart clothes is a power source to operate the sensors, electronics, and other components requiring electric power for proper operation. To solve the problem of limited lifetime and the need for periodic recharge of the battery, energy harvesters, which convert mechanical energy into electrical energy and function as semipermanent power sources without the need for an external battery, are being studied [27,28,29,30,31,32,33].

Recently, several devices having sensors and energy harvesters in a single platform were reported. These devices sense body activity or environmental signals and, at the same time, generate electric power from the human body [29,30,31,32,33,34]. Ahn et al. reported on a woven-structured power-generating tactile sensor array in which a piezoelectric energy harvester and tactile sensors were integrated in a single device [29]. In addition, Saha et al. proposed an electromagnetic generator from human motion and proposed a structure that can be applied to a body-worn sensor [33]. We have also reported a triboelectric and piezoresistive property of a single hollow tube structure with surface modification [34]. That report presented that the electrode layers for energy harvester could be utilized as a piezoresistive sensing layers.

The triboelectric energy harvester has been widely developed and utilized in converting mechanical energy into electrical energy from periodic contact and separation between materials having different charge affinities. Triboelectric energy harvesters have several advantages including easy fabrication, cost effectiveness, high energy conversion efficiency, and the possibility of using a variety of materials. In addition, since they are easily implemented by using polymer-based or flexible materials, they are suitable for smart-clothes applications requiring self-power generation [35,36,37,38,39,40]. 

Thus, in this work, we propose a device composed of resistive strain sensors, capacitive tactile sensors, and a triboelectric energy harvester in a single platform. The proposed device has a woven textile structure that provides an expandable tactile and strain sensor array. In addition, the output power as a triboelectric energy harvester has been increased by applying surface modifications of the materials using self-assembly monolayer (SAM) coating [34,41]. 

## 2. Principle

**Figure 1:**
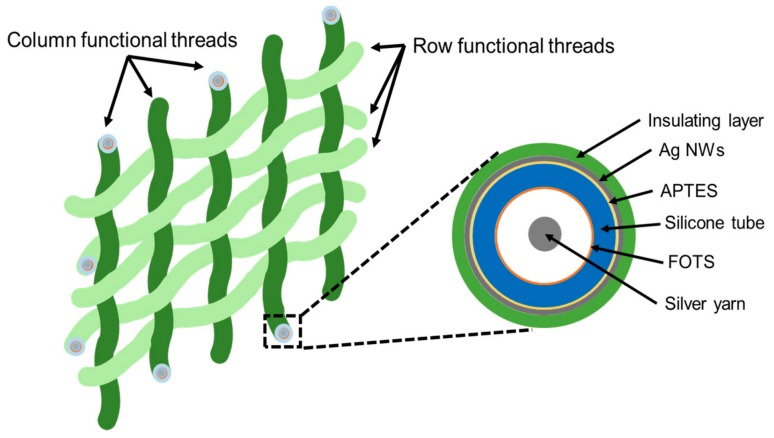
Schematic of proposed device.

Figure 1 shows a top view and cross-sectional view of the proposed device. The device has a woven textile structure consisting of functional weft and warp threads. As shown in the cross-sectional view, each functional thread is composed of a flexible tube with silver yarn inside the tube. Silver nanowires (Ag NWs) are coated on the outer surface of the tube, and an insulating layer, which is a mixture of polydimethylsiloxane (PDMS) and EcoFlex, is coated on it. The Ag NWs layer shows electrical conductivity and piezoresistivity as well. Thus, we utilized this layer as a conductive electrode for signal and power transfer as well as a sensing layer during the strain sensor operation of the device. The inner surface of the tube is coated with 1H, 1H, 2H, 2H-perfluorrooctyltrichlorosilane (FOTS) to have a high output power during the energy-harvesting operation of the device by changing the triboelectric property of the surface.

The proposed device can be operated in three different modes. First, as a triboelectric energy harvester, it can generate electric power from contact between the inner surface of the tube and the silver yarn when a pressurizing force is applied. Second, the proposed device can detect the magnitude and position of a vertical touch force as a tactile sensor array. Third, as a strain sensor, a lateral stretching force and its location can be detected by measuring the resistance of each thread. In this section, we will discuss the operating principles of the device in the three different modes.

### 2.1. Energy-Harvesting Mode

**Figure 2:**
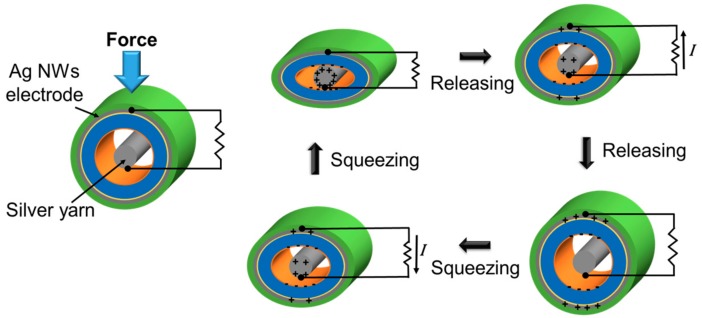
Cross-sectional view of the device to explain energy-harvesting operation during repetitive contact-separation motion.

Figure 2 shows the charge generation mechanism of a single functional thread of the proposed device when it is used in the energy-harvesting mode. The energy generation process can be explained by the triboelectrification and electrostatic induction effect. The silver yarn inserted in the tube either touches the wall or is separated from the wall depending on the position and curvature of the thread. If the silver yarn is separated from the wall as shown in Figure 2, initially, each surface of the tube and silver yarn are electrically neutral. When a force is applied to the thread and the tube and silver yarn are in contact, electrons are injected into the FOTS-coated surface from the silver yarn because the FOTS-coated surface has a more negative property than silver in the triboelectric table [42]. This leads to positive charges on the silver yarn and negative charges on the surface of the tube. For the region where the silver yarn is initially in contact to the wall, the charge transfer still occurs through the additional contact between the silver yarn and the tube on the other side of the silver yarn when a squeezing force is applied.

As silver yarn is separated from the inner surface of the tube after the force is removed, the positive charges flow from the silver yarn to the Ag electrode coated on the tube, which results in current flow. When the pressurizing force is applied again, the positive charges on the tube electrode move to the silver yarn, resulting in current flow in the reverse direction, as shown in the figure. Similarly, the charge transfer and resultant current flow can be obtained from the multiple threads woven in the textile structure when a pressurizing force is applied to some region of the structure and makes the silver yarn contact the tube. Therefore, alternating output voltage and current are generated as the devices are repeatedly pushed and released. 

### 2.2. Capacitive Tactile Sensor Mode

**Figure 3:**
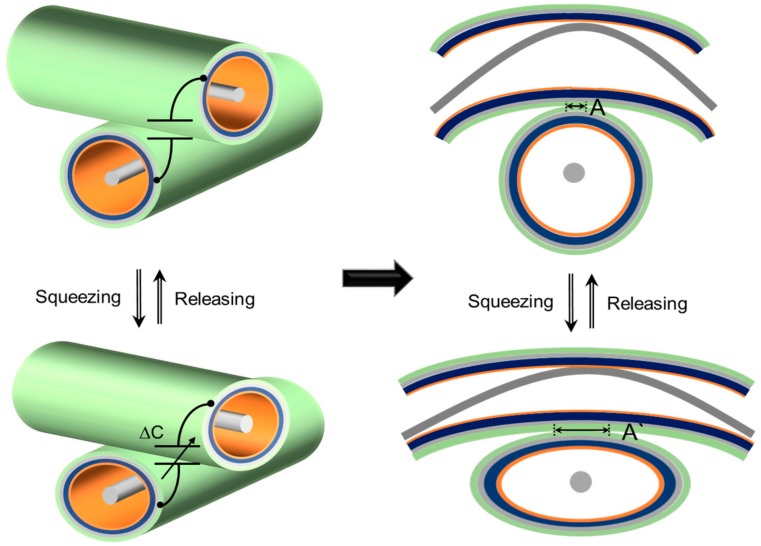
Magnified view and cross-sectional view of device explaining capacitive sensor mode.

The proposed device can be operated as a capacitive tactile sensor to monitor the magnitude and position of a tactile force applied to it. This tactile force in the normal direction can be measured by reading the capacitance formed between a weft and a warp functional thread. Figure 3 shows three-dimensional and cross-sectional views of the intersection of the weft and warp threads. The outer surface of the silicone tube is coated with Ag NWs and an insulating layer. Therefore, the capacitance is formed between the Ag NWs layers deposited on the weft and warp functional thread. When pressure is applied in the vertical direction, the elastic threads are compressed, and consequently, the overlap area between each electrode increases, which increases the capacitance accordingly. 

Because the overlap area and consequently the capacitance increases as the pressure increases, the magnitude of the tactile force can be measured by reading the capacitance value. In addition, the touch position can be found by sequentially measuring the capacitances formed by each set of warp and weft threads. In this study, we used a total of 10 functional threads—five in the row direction and five in the column direction—resulting in 5 × 5 capacitive cells on the device. 

### 2.3. Resistive Strain Sensor Mode

**Figure 4:**
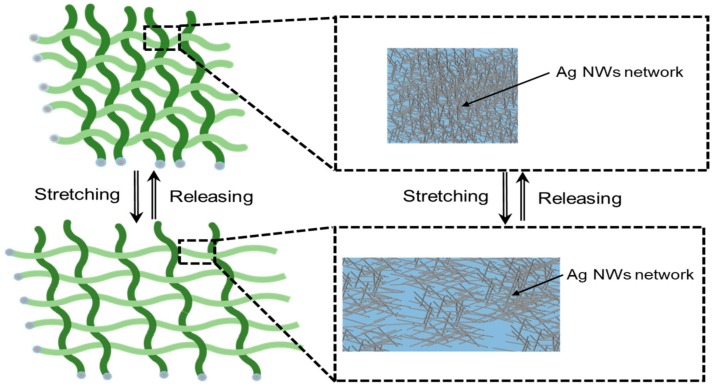
Schematic view of Silver nanowires (Ag NWs) layer before and after applying stretching force.

Figure 4 shows the operation of the proposed device in resistive-strain-sensor mode. Each of the horizontal and vertical tubes is coated with Ag NWs, which become a piezoresistive layer [43,44,45]. When stretched, the resistance between the ends of each functional thread increases as the connections between the silver nanowires on the electrode layer break. In our design, the magnitude and direction of strains applied on the tubes are easily monitored by sequential measurements of the resistances of each functional thread. In this manner, the unwanted signals affecting the sensor output such as triboelectric effect coming from the friction between row and column threads can be minimized. In this work, we implemented the device in 5 × 5 tubes in the row and column directions.

## 3. Fabrication

**Figure 5:**
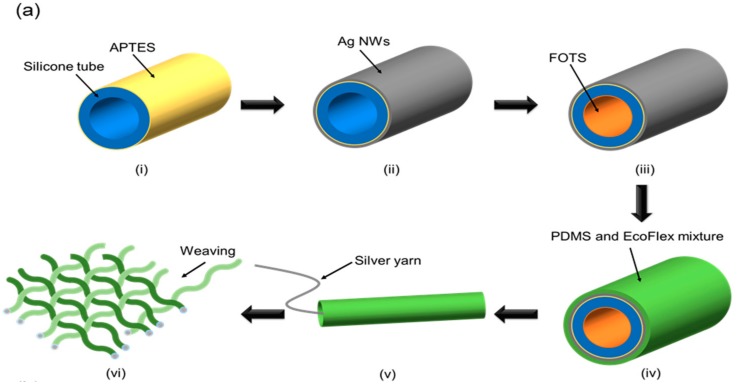

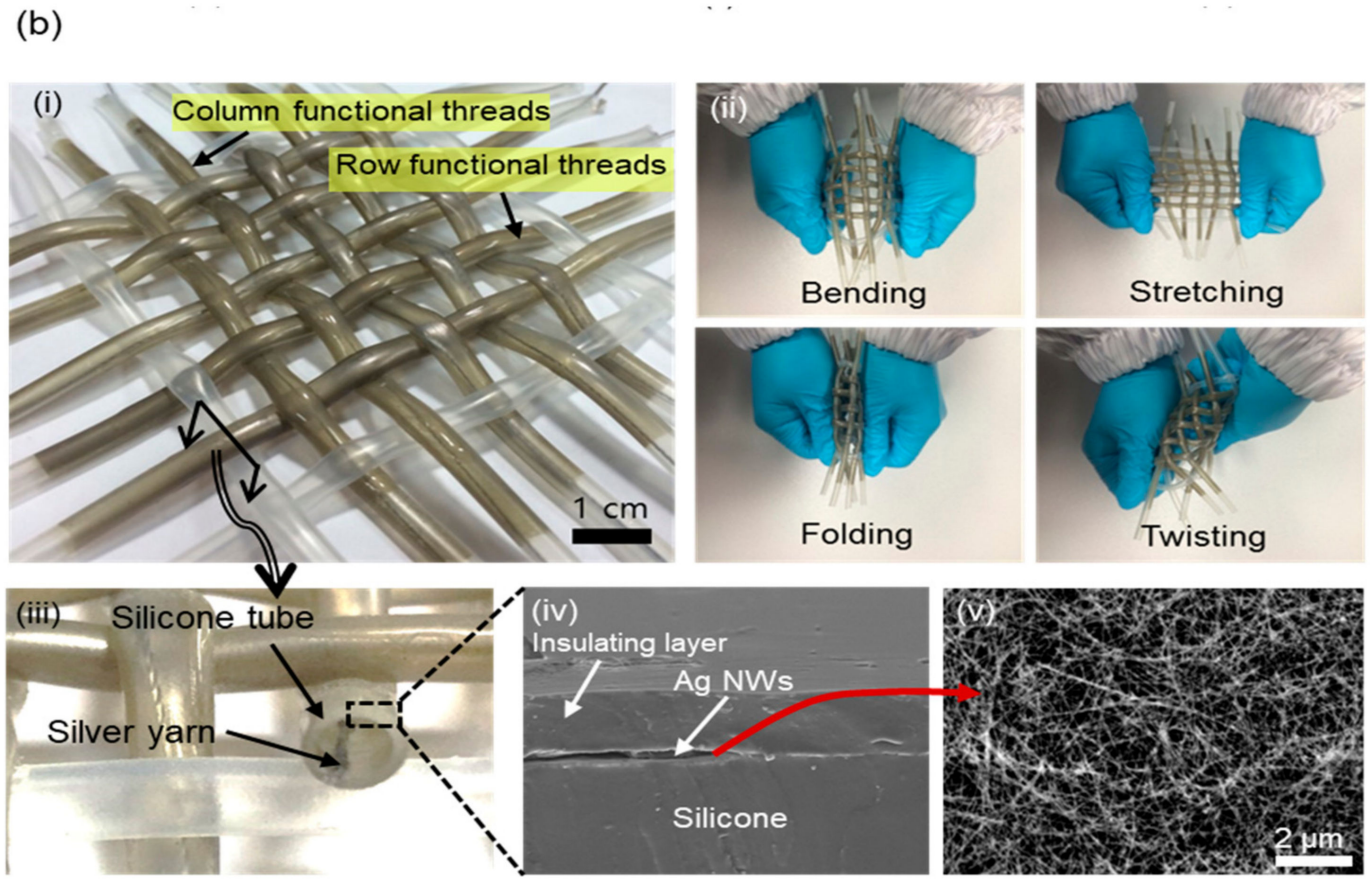
(**a**) Fabrication steps: (i) 3-aminipropyltriethoxysilane (APTES) coating on outer surface of tube. (ii) Ag NWs coating on surface. (iii) FOTS coating on inner surface. (iv) Coating of polydimethylsiloxane (PDMS)-EcoFlex layer. (v) Inset of silver yarn. (vi) Weaving threads into textile structure. (**b**) Photograph of fabricated device: (i) Fabricated device. (ii) Bending, stretching, folding, twisting test using device. (iii) Cross-sectional view of a functional thread. (iv) Scanning electron microscope (SEM) image of the cross-section of the functional thread. (v) SEM image of the Ag NWs layer coated on the silicone tube.

Figure 5a shows the fabrication process of the proposed device. First, the silicone tubes were cleaned by soaking in ethanol for 1 min, followed by treatment in oxygen plasma (50 W, 40 sccm, 120 s) to develop hydroxyl groups on the outer surface of the tube. Then, the tube was immersed in 3-wt % 3-aminipropyltriethoxysilane (APTES) solution for 3 h to enhance the adhesion of Ag NWs on the surface of the silicone tube by forming amine group [46]. Next, an Ag NWs layer was spray-coated on the outer surface of the tube using 10 wt % of ethanol-based Ag NWs solution (DT-AGNW-N30-EOH, Ditto Technology). The air brush of the spray gun (Air Master PRO-03 model) was set at 20 psi with a feed rate of 45 mL/h.

Then, the tube was heat-treated at 150 °C for 20 min to cure the Ag NWs layer. Next, a PDMS (Sylgard 184, Dow Corning, Midland, MI, USA) and EcoFlex (EcoFlex 0010, SMOOTH-ON, Macungie, PA, USA) mixture with a weight mixing ratio of 1:9 was coated on the Ag NWs layer as an insulator. This insulator coating was done by a rolling process, as shown in Figure 5a. Next, silver-coated yarn was inserted into the tube after FOTS coating on the inner surface of the tube in a desiccator. Finally, the proposed device was weaved into the woven textile structure by crossing 10 threads. 

Figure 5b shows a photograph of the fabricated device. The size of the device is 6 × 6 cm^2^, and it can easily be bent, twisted, stretched, and folded because the device was fabricated with fully flexible materials.

## 4. Results and Discussion

### 4.1. Energy-Harvesting Mode

**Figure 6:**
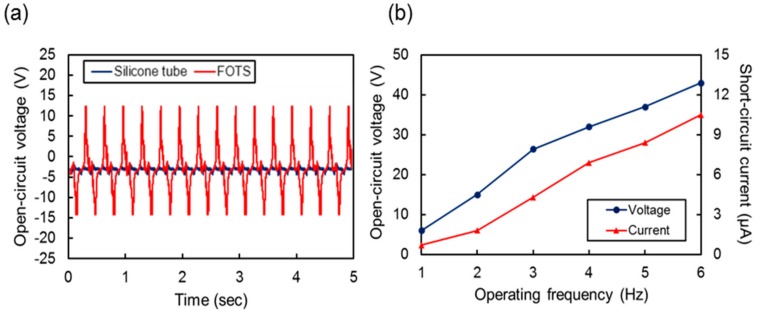
(**a**) Real time profiles of open-circuit output voltages measured at frequency of 3 Hz with vertical force of 10 N with and without 2H-perfluorrooctyltrichlorosilane (FOTS) coating. (**b**) Peak-to-peak open-circuit voltage and short-circuit current from single thread at various operation frequencies.

Figure 6a shows the output properties of the prepared single functional thread when it is pressed and released repeatedly by a stamp with 10 N of pressing force. The actuation frequency was 3 Hz in this experiment. The open-circuit output voltage was 2.3 V without the FOTS coating. However, by coating the inner surface of the tube with FOTS, we could obtain an output of 27.7 V, which is 12 times greater than the output without the FOTS coating, because FOTS, which is composed of fluorine atoms, provides a higher electron affinity compared to a bare silicone surface. 

Figure 6b shows the open-circuit output voltage and short-circuit current from a single thread operated at various actuation frequencies. We used a shaker (LDS V406, Bruel & Kjaer, Naerum, Denmark) to apply a repeated pressurizing force to the thread at various vibration frequencies. Both the output voltage and current increased with the actuation frequency. The maximum output voltage and current were measured as 43 V and 9.9 μA, respectively, at 6 Hz. As shown in the graph, the voltage and current increased with the frequency because the pressurizing force increased as the moving frequency and acceleration increased [47].

**Figure 7:**
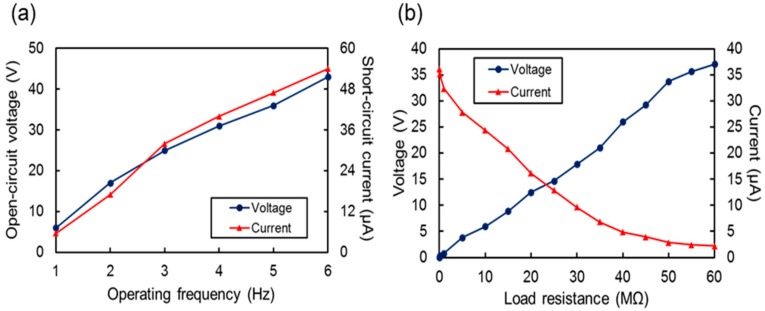

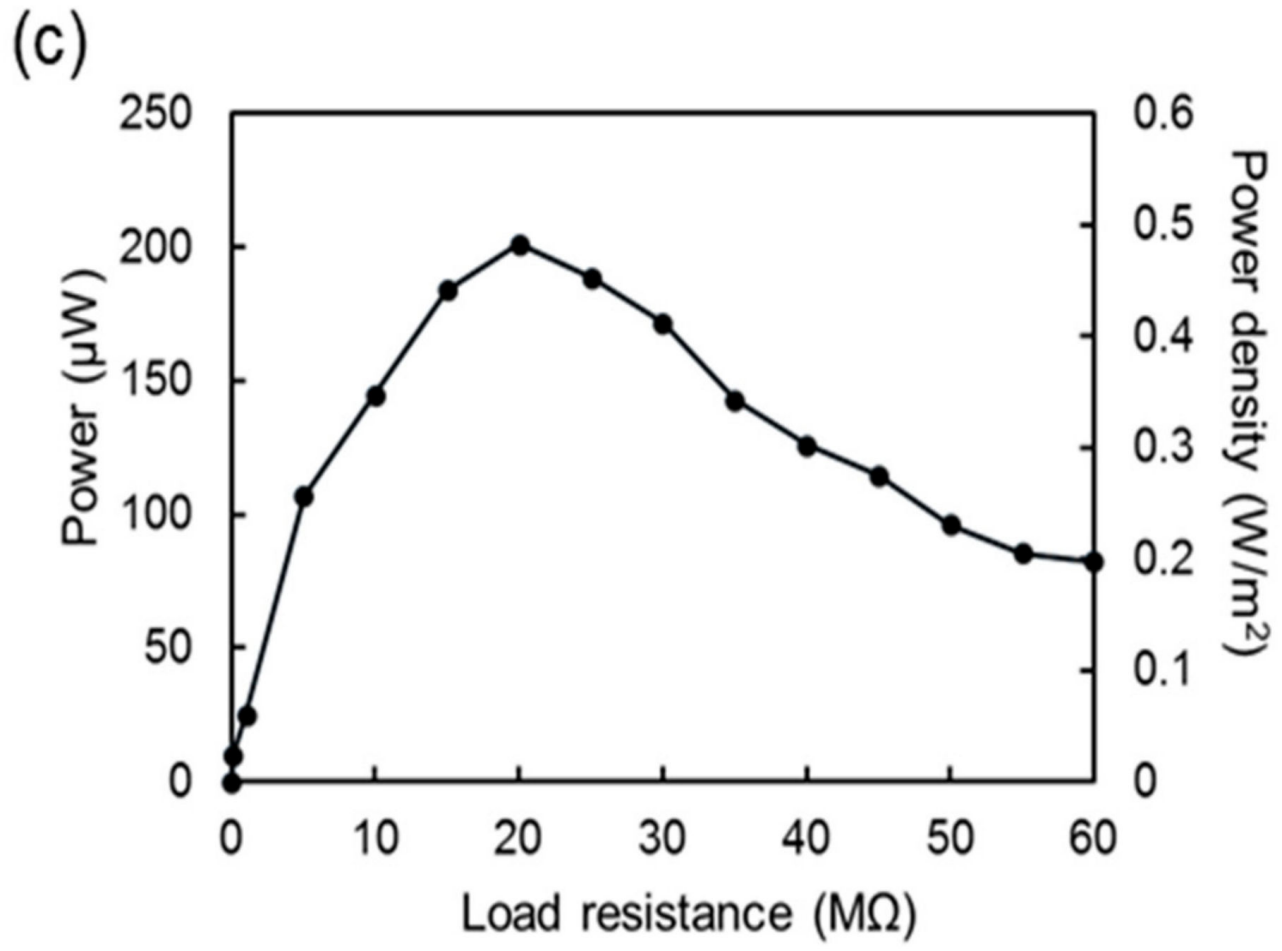
(**a**) Peak-to-peak open-circuit voltage and short-circuit current from 5 × 5 arrays at various frequencies. (**b**) Out voltage and current from 5 × 5 arrays for various load resistances. (**c**) Output power and power density for various load resistances.

The output properties of a complete device having 5 × 5 row and column threads in a woven structure are shown in Figure 7. All 10 threads were set in a parallel connection electrically. Figure 7a shows the open-circuit voltage and short-circuit current. The output voltage did not make a substantial difference compared to the value from a single thread in Figure 6 because the threads were connected in parallel. However, the short-circuit current was increased to 52 μA at 6 Hz, which is five times larger compared to the output from a single thread. Theoretically, this is supposed to produce 10 times more output current than a single thread. However, in this experiment, the output might be decreased because the pressurizing and triboelectric contacts were not perfectly even to all threads. Consequently, the signals from each thread were not perfectly synchronized. 

Figure 7b shows the peak-to-peak output voltage and current measured at various load resistances. In this work, we measured the voltage and current across the load using an oscilloscope and a low-noise current preamplifier (SR0570, Stanford Research Systems, Sunnyvale, CA, USA), respectively, to determine the optimal load resistance. As the load resistance increased, the output current decreased because the source current was divided between the internal impedance and load resistor by the magnitude ratio of the parallel-connected impedances. By contrast, the output voltage increased with the resistance. The load resistance was optimal when the product of the voltage and current was at its maximum value. The relationship between the load resistance and the peak-to-peak output power is shown in Figure 7c for an operating frequency of 3 Hz and applied force of 10 N, respectively, where the maximum power was measured as 201 μW and 0.48 W/m^2^, respectively, at a load resistance of 20 MΩ.

### 4.2. Capacitive Tactile Sensor Mode

**Figure 8:**
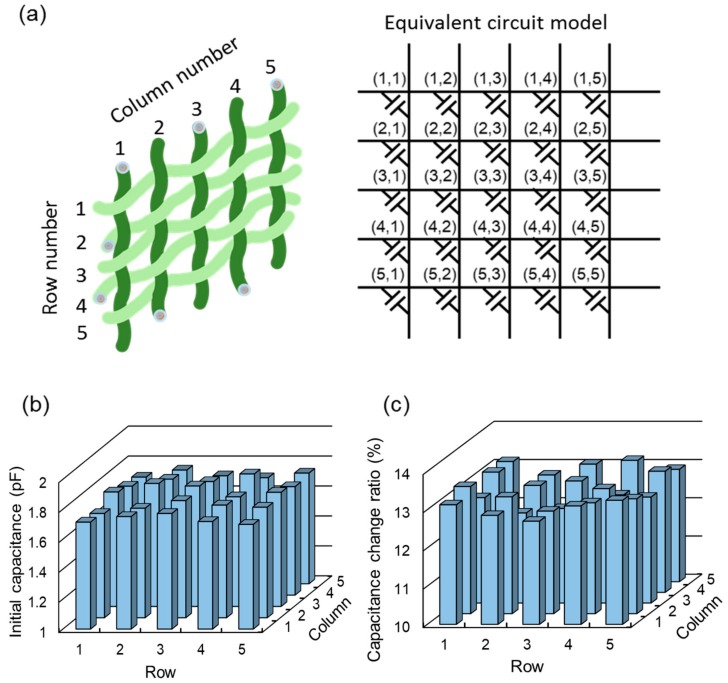
(**a**) Equivalent circuit model of the device in capacitive tactile sensor mode. (**b**) Initial capacitances of sensor composed of 25 capacitive cells. (**c**) Capacitance change ratio after applying vertical force of 2 N.

As described in Section 2.2, the proposed device can monitor the location and magnitude of the vertical pressurizing force by measuring the capacitances formed at the crossing positions of the row and column threads. As shown in Figure 8a, the capacitive sensing cells are formed at the intersections of each row and column functional thread. The initial capacitances of the 5 × 5 capacitive cells in the fabricated device are shown in Figure 8b. We measured the capacitance value using an LCR meter (4284A, Agilent, Santa Clara, CA, USA). The initial average capacitance of a total of 25 cells was 1.789 pF with a standard deviation of 30 fF. The slight variation between cells might come from the nonuniform thickness of the insulating layer and variations in the gap distance between cells. These are inevitable fabrication errors resulting from manual assembly steps during device fabrication. This initial offset can be minimized by applying a compensation technique to the readout circuit or a signal processing module in practical applications. Figure 8c shows the capacitance values after applying a vertical force of 2 N to each cell. The capacitance was increased by 12.8% on average with a standard deviation of 40 fF.

**Figure 9:**
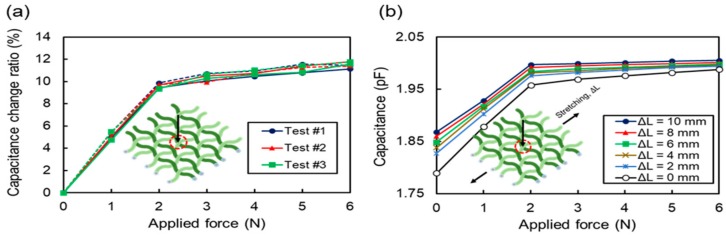
(**a**) Sensitivity and hysteresis response measured at a (3, 3) capacitive cell while increasing and decreasing vertical pressurizing force for three repeated applications. The measured capacitive cell was marked with a red circle in the inset. The arrow in the inset shows the direction of the applied vertical force. (**b**) Capacitance of (3, 3) capacitive cell measured at various vertical forces under various lateral stretching lengths.

The sensitivity of a single cell was measured and is depicted in Figure 9a while applying various vertical forces to a specific cell by using a force gauge (HF-50, JISC, Bristol, UK). The capacitance increases as the applied force increases because the contact area between the row and column threads increases with force. The initial capacitance was measured as 1.79 pF when the device was not pushed. The capacitance increased as the normal force increased, and was measured as 1.97 pF at a force of 2 N. The sensitivity of the capacitive cell was approximately 4.72%/N in a force range of 2 N. However, the capacitive cell became less sensitive to a normal force larger than approximately 2 N because the hollow tubes were almost collapsed and were barely deformed. The sensitivity in this regime is approximately 0.24%/N in a force range from 2 N to 6 N. 

In this experiment, to determine the hysteresis of the sensor, the capacitance was measured while increasing and decreasing the applied force on the tactile sensor three times repeatedly. The maximum difference between the capacitance values measured during force increment and decrement was 6 fF, which is 0.31% of the initial capacitance. This low hysteresis is a good characteristic for sensor applications. 

Figure 9b shows the capacitance values measured from a single cell of the tactile sensor array for the applied force while applying various lateral forces to extend the threads by 2, 4, 6, and 10 mm. In this experiment, the data was obtained from the capacitive cell formed at the intersection point of the third row and third column threads of the device. The initial capacitance, where no vertical force was applied, increased as the lateral stretching length increased because the distance between the top and bottom electrodes decreased as the device was laterally stretched.

### 4.3. Resistive Strain Sensor Mode

**Figure 10:**
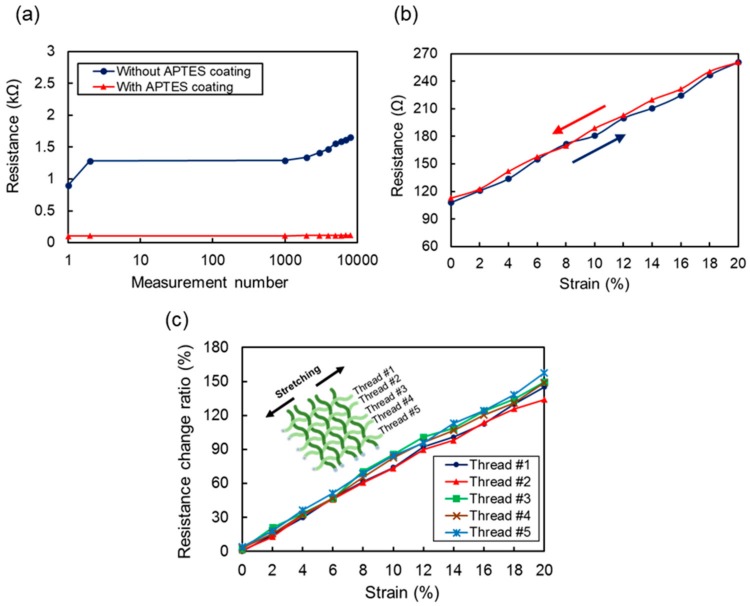
(**a**) Comparison of durability test using devices with and without APTES coating on hollow tube when lateral force was applied up to 20% strain for 8000 times. (**b**) Hysteresis response of single thread measured while lateral force was applied to increase (navy color) and decrease (red color) the strain gradually. (**c**) Sensitivity variation between five functional threads.

The stretching or in-plane strain can be measured by reading the resistance change in each column and row thread. Figure 10a shows the resistance of a single thread in the releasing state during repeated stretching and releasing actuations of up to 8000 cycles. In this experiment, the stretching length of the thread was fixed to 20% of its initial length. Two different threads were tested. One was a thread coated with APTES on the outer surface of the silicone tube before Ag NWs deposition, and no pretreatment was used for the other thread. As shown in the graph, the initial resistance values with and without APTES were 104 Ω and 890 Ω for each. After we pulled and released the thread for 8000 cycles, the resistance of the device without the APTES coating was increased to 1.65 kΩ, which is an 86% increment. However, little increase in resistance was observed for the APTES-coated thread. In this experiment, it is clear that the stability of the Ag NWs layer on a silicone surface can be dramatically increased by applying APTES as an adhesion layer.

The piezoresistive property of an APTES-coated thread was measured using a motorized linear actuator to apply uniaxial tensile strain. Figure 10b shows the resistance change when the thread is pulled up to 20% and released to its initial length. We can see that the resistance increased and decreased linearly as the strain changed. The gauge factor was about 12.1, which is much larger than that of the conventional metallic strain gauge. In addition to good linearity and sensitivity, the hysteresis of the fabricated thread as a strain sensor was quite low, which was a maximally 4.6% difference during stretching and releasing. The resistance change of the five threads in the fabricated 5 × 5 woven array device was also measured while applying a tensile strain to the threads, of up to 20%. As shown in Figure 10c, the sensitivity variation between threads was very small, which can be easily compensated in a readout circuit or through signal processing.

## 5. Conclusions

In this research, we proposed and demonstrated a power-generating multi-sensor array that can be operated as a triboelectric energy harvester, a capacitive tactile sensor, and a resistive strain sensor. This device is able to detect signals through human movement and generate power by energy harvesting in a single device. The proposed device was implemented by a simple fabrication process such as dip coating, spray coating, or vacuum coating. In addition, the proposed device, which consists of stretchable and flexible materials including hollow tubes, Ag NWs layers, and PDMS and EcoFlex mixtures, is easily bendable, stretchable, foldable, and twistable and has excellent durability. 

The prototype device was made up of five-row and five-column functional threads, resulting in a 5 × 5 sensor array in an area of 6 × 6 cm^2^. When operated as an energy harvester, the maximum power and power density were measured as 210 μW and 0.48 W/m^2^, respectively, at a 20-MΩ load resistance. In addition, it was demonstrated that the capacitance output increased with a vertical pressurizing force as a tactile sensor and the resistance output increased with a lateral stretching force as a strain sensor. The proposed device is expected to be used in a variety of fields to monitor human motions and activities because the device was fabricated in a woven structure similar to actual clothes and can be extended to a large scale.

## References

[1] Moran S., Nishida T., Nakata K. (2013). Perceptions of a wearable ubiquitous monitoring device. IEEE Technol. Soc. Mag..

[2] Windmiller J.R., Wang J. (2013). Wearable electrochemical sensors and biosensors: A review. Electroanalysis.

[3] Bandodkar A.J., Jia W., Ramírez J., Wang J. (2015). Biocompatible Enzymatic Roller Pens for Direct Writing of Biocatalytic Materials: “Do-it-Yourself” Electrochemical Biosensors. Adv. Healthc. Mater..

[4] Barro S., Ruiz R., Cabello D., Mira J. (1989). Algorithmic sequential decision-making in the frequency domain for life threatening ventricular arrhythmias and imitative artefacts: A diagnostic system. J. Biomed. Eng..

[5] Chi Y.M., Jung T.P., Cauwenberghs G. (2010). Dry-contact and noncontact biopotential electrodes: Methodological review. IEEE Rev. Biomed. Eng..

[6] Kim D.S., Hwang T.H., Song J.Y., Park S.H., Park J., Yoo E.S., Lee N.K., Park J.S. (2016). Design and Fabrication of Smart Band Module for Measurement of Temperature and GSR (Galvanic Skin Response) from Human Body. Proced. Eng..

[7] Amft O., Wahl F., Ishimaru S., Kunze K. (2015). Making regular eyeglasses smart. IEEE Pervasive Comput..

[8] Kim J., Kim M., Lee M.S., Kim K., Ji S., Kim Y.T., Park J., Na K., Bae K.W., Kim H.K. (2017). Wearable smart sensor systems integrated on soft contact lenses for wireless ocular diagnostics. Nat. Commun..

[9] Guinovart T., Bandodkar A.J., Windmiller J.R., Andrade F.J., Wang J. (2013). A potentiometric tattoo sensor for monitoring ammonium in sweat. Analyst.

[10] Kong K., Tomizuka M. (2009). A gait monitoring system based on air pressure sensors embedded in a shoe. IEEE/ASME Trans. Mechatron..

[11] Syduzzaman M., Patwary S.U., Farhana K., Ahmed S. (2015). Smart textiles and nano-technology: A general overview. J. Text. Sci. Eng..

[12] Özdemir H., Kılınç S. (2015). Smart woven fabrics with portable and wearable vibrating electronics. Autex Res. J..

[13] Gong S., Schwalb W., Wang Y., Chen Y., Tang Y., Si J., Shirinzadeh B., Cheng W. (2014). A wearable and highly sensitive pressure sensor with ultrathin gold nanowires. Nat. Commun..

[14] Yamada T., Hayamizu Y., Yamamoto Y., Yomogida Y., Izadi-Najafabadi A., Futaba D.N., Hata K. (2011). A stretchable carbon nanotube strain sensor for human-motion detection. Nat. Nanotechnol..

[15] Findlow A., Goulermas J.Y., Nester C., Howard D., Kenney L.P.J. (2008). Predicting lower limb joint kinematics using wearable motion sensors. Gait Posture.

[16] Yun Y.J., Hong W.G., Choi N.J., Kim B.H., Jun Y., Lee H.K. (2015). Ultrasensitive and highly selective graphene-based single yarn for use in wearable gas sensor. Sci. Rep..

[17] Yang J., Wei D., Tang L., Song X., Luo W., Chu J., Gao T., Shi H., Du C. (2015). Wearable temperature sensor based on graphene nanowalls. RSC Adv..

[18] Arshak K., Morris D., Korostynska O., Jafer E., Arshak A., Harris J., Clifford S., Lyons G. (2004). Novel silicone-based capacitive pressure sensors with high sensitivity for biomedical applications. E-Polymer.

[19] Ohka M., Kobayashi H., Takata J., Mitsuya Y. (2008). An experimental optical three-axis tactile sensor featured with hemispherical surface. J. Adv. Mech. Des. Syst. Manuf..

[20] Takashima K., Horie S., Mukai T., Ishida K., Matsushige K. (2008). Piezoelectric properties of vinylidene fluoride oligomer for use in medical tactile sensor applications. Sens. Actuators A Phys..

[21] Hsieh M.C., Fang Y.K., Ju M.S., Chen G.S., Ho J.J., Yang C.H., Wu P.M., Wu G.S., Chen T.Y.F. (2001). A contact-type piezoresistive micro-shear stress sensor for above-knee prosthesis application. J. Microelectromech. Syst..

[22] Lee J., Kwon H., Seo J., Shin S., Koo J.H., Pang C., Son S., Kim J.H., Jang Y.H., Kim D.E. (2015). Conductive Fiber-Based Ultrasensitive Textile Pressure Sensor for Wearable Electronics. Adv. Mater..

[23] Hasegawa Y., Shikida M., Ogura D., Suzuki Y., Sato K. (2008). Fabrication of a wearable fabric tactile sensor produced by artificial hollow fiber. J. Micromech. Microeng..

[24] Wang C., Li X., Gao E., Jian M., Xia K., Wang Q., Xu Z., Ren T., Zhang Y. (2016). Carbonized silk fabric for ultrastretchable, highly sensitive, and wearable strain sensors. Adv. Mater..

[25] Li Y., Samad Y.A., Taha T., Cai G., Fu S.Y., Liao K. (2016). Highly flexible strain sensor from tissue paper for wearable electronics. ACS Sustain. Chem. Eng..

[26] Park S.J., Kim J., Chu M., Khine M. (2016). Highly Flexible Wrinkled Carbon Nanotube Thin Film Strain Sensor to Monitor Human Movement. Adv. Mater. Technol..

[27] Park J., Yun K.S. Hybrid energy harvester based on piezoelectric and triboelectric effects. Proceedings of the 2016 IEEE 29th International Conference on Micro ElectroMechnical Systems (MEMS).

[28] Jabbar H., Song Y.S., Jeong T.T. (2010). RF energy harvesting system and circuits for charging of mobile devices. IEEE Trans. Consum. Electron..

[29] Song S., Yun K.S. (2015). Design and characterization of scalable woven piezoelectric energy harvester for wearable applications. Smart Mater. Struct..

[30] Ahn Y., Song S., Yun K.S. (2015). Woven flexible textile structure for wearable power-generating tactile sensor array. Smart Mater. Struct..

[31] Yun D., Park J., Yun K.S. (2015). Highly stretchable energy harvester using piezoelectric helical structure for wearable applications. Electron. Lett..

[32] Kim M., Yun K.S. (2017). Helical Piezoelectric Energy Harvester and Its Application to Energy Harvesting Garments. Micromachines.

[33] Saha C.R., O’donnell T., Wang N., McCloskey P. (2008). Electromagnetic generator for harvesting energy from human motion. Sens. Actuators A Phys..

[34] Kim K., Yun K.-S. Triboelectric Energy Harvester in Hollow Tube Structure and Its Sensor Property. Proceedings of the 2017 IEEE 12th International Conference on Nano/Micro Engineered and Molecular Systems (NEMS).

[35] Shi M., Wu H., Zhang J., Han M., Meng B., Zhang H. (2017). Self-powered wireless smart patch for healthcare monitoring. Nano Energy.

[36] Liu L., Pan J., Chen P., Zhang J., Yu X., Ding X., Wang B., Sun X., Peng H. (2016). A triboelectric textile templated by a three-dimensionally penetrated fabric. J. Mater. Chem. A.

[37] Seung W., Gupta M.K., Lee K.Y., Shin K.S., Lee J.H., Kim T.Y., Kim S., Lin J., Kim J.H., Kim S.W. (2015). Nanopatterned textile-based wearable triboelectric nanogenerator. ACS Nano.

[38] Pu X., Li L., Song H., Du C., Zhao Z., Jiang C., Cao G., Hu W., Wang Z.L. (2015). A self-charging power unit by integration of a textile triboelectric nanogenerator and a flexible lithium-ion battery for wearable electronics. Adv. Mater..

[39] Cui N., Liu J., Gu L., Bai S., Chen X., Qin Y. (2015). Wearable triboelectric generator for powering the portable electronic devices. ACS Appl. Mater. Interfaces.

[40] Lee S., Ko W., Oh Y., Lee J., Baek G., Lee Y., Sohn J., Cha S., Kim J., Park J. (2015). Triboelectric energy harvester based on wearable textile platforms employing various surface morphologies. Nano Energy.

[41] Song G., Kim Y., Yu S., Kim M.O., Park S.H., Cho S.M., Velusamy D.B., Cho S.H., Kim K.L., Kim J. (2015). Molecularly engineered surface triboelectric nanogenerator by self-assembled monolayers (METS). Chem. Mater..

[42] Shin S.H., Bae Y.E., Moon H.K., Kim J., Choi S.H., Kim Y., Yoon H.J., Lee M.H., Nah J. (2017). Formation of Triboelectric Series via Atomic Level Surface Functionalization for Triboelectric Energy Harvesting. ACS Nano.

[43] Wei Y., Chen S., Dong X., Lin Y., Liu L. (2017). Flexible piezoresistive sensors based on “dynamic bridging effect” of silver nanowires toward graphene. Carbon.

[44] Yan C., Wang J., Kang W., Cui M., Wang X., Foo C.Y., Chee K.J., Lee P.S. (2014). Highly stretchable piezoresistive graphene–nanocellulose nanopaper for strain sensors. Adv. Mater..

[45] Wei Y., Chen S., Li F., Lin Y., Zhang Y., Liu L. (2015). Highly stable and sensitive paper-based bending sensor using silver nanowires/layered double hydroxides hybrids. ACS Appl. Mater. Interfaces.

[46] Lee M.H., Brass D.A., Morris R., Composto R.J., Ducheyne P. (2005). The effect of non-specific interactions on cellular adhesion using model surfaces. Biomaterials.

[47] Kim D., Jeon S.B., Kim J.Y., Seol M.L., Kim S.O., Choi Y.K. (2015). High-performance nanopattern triboelectric generator by block copolymer lithography. Nano Energy.

